# Case report of left renal cell carcinoma with long standing retained common bile duct stent and secondary choledocholithiasis: a single-stage multidisciplinary surgical management in low resource setting

**DOI:** 10.1093/jscr/rjaf783

**Published:** 2025-10-02

**Authors:** Manzoor Ahmad, Azeem Farooqui, Ahmad Sadiq, Wasif M Ali, Mazher Alam

**Affiliations:** Department of Surgery, JNMCH, AMU, Aligarh 202002, Uttar Pradesh, India; Department of Surgery, JNMCH, AMU, Aligarh 202002, Uttar Pradesh, India; Department of Surgery, JNMCH, AMU, Aligarh 202002, Uttar Pradesh, India; Department of Surgery, JNMCH, AMU, Aligarh 202002, Uttar Pradesh, India; Department of Surgery, JNMCH, AMU, Aligarh 202002, Uttar Pradesh, India

**Keywords:** renal cell carcinoma, choledocholithiasis, retained common bile duct stent, nephrectomy, multidisciplinary surgery

## Abstract

A 65-year-old male presented with a left renal mass and a retained common bile duct stent from a previous endoscopic retrograde cholangiopancreatography 2 years ago for choledocholithiasis. Imaging confirmed a lower pole renal lesion with features of renal cell carcinoma (RCC) and dilated biliary tree with sludge and a retained stent. The patient underwent single-stage surgery—left radical nephrectomy and open choledochoduedenotomy—for removal of the stent and biliary clearance through midline approach. Histopathology confirmed chromophobe RCC. This case illustrates the feasibility of patient-centred manner simultaneous surgical treatment for dual-system pathologies in a low resource setting and.

## Introduction

Renal cell carcinoma (RCC) is the most common primary malignancy of the kidney, accounting for nearly 90% of all renal cancers in adults. It originates from the renal tubular epithelium and is known for its unpredictable clinical behaviour and resistance to conventional chemotherapy and radiotherapy [1]. Traditionally described by the triad of haematuria, flank pain, and a palpable mass, this classic presentation is now seen in ˂10% of cases, due to the increasing use of imaging for unrelated abdominal complaints, which leads to incidental detection of many renal tumours [2]. Despite advances in diagnostic imaging and surgical management, a considerable proportion of RCC cases still present at an advanced stage, often posing significant treatment challenges.

Choledocholithiasis refers to the presence of gallstones in the common bile duct (CBD) and is a common complication in patients with gallstone disease. It can present acutely with symptoms such as right upper quadrant pain, jaundice, or fever, or remain asymptomatic for extended periods [3]. The standard approach to management includes endoscopic retrograde cholangiopancreatography (ERCP), which allows for stone removal and CBD drainage. In cases where immediate clearance is not feasible, plastic CBD stents are placed to relieve obstruction and facilitate biliary flow. These stents are ideally placed temporary and should be removed or exchanged within 3–6 months [4]. However, in third-world settings, these same patients are lost to follow-up or unaware of the need for stent removal. Stents can remain in situ for prolonged periods. This leads to significant complications including cholangitis, stone formation around the stent, and even secondary biliary cirrhosis [5].

The simultaneous occurrence of RCC and choledocholithiasis in the same patient is rare. The clinical scenario becomes even more complex when there is associated history of a retained CBD stent for an extended period, as seen in this case. Each condition requires a careful and often time-sensitive therapeutic approach. This case highlights the crucial role of multidisciplinary collaboration involving urologists, GI surgeons, radiologists, and anaesthesiologists in the management of patients with multiple organ surgical pathologies.

## Case report

A 65-year-old male presented with vague abdominal discomfort and a palpable lump in the left flank. There was no history of haematuria or constitutional symptoms. Notably, the patient had undergone ERCP with CBD stenting somewhere else for choledocholithiasis 2 years back but was lost to follow-up.

**Figure 1:**
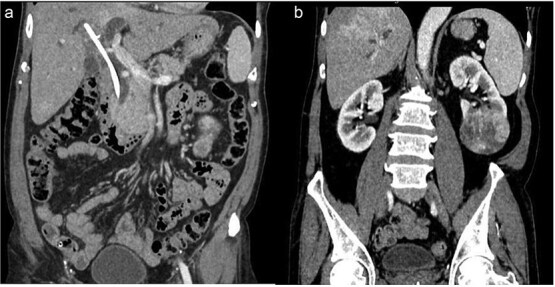
(a) Retained CBD stent. (b) Necrotic mass at lower pole of left kidney.

Examination revealed mild right upper quadrant tenderness and lump in left lumbar region. Liver function tests showed mildly elevated transaminases. Ultrasonography and contrast-enhanced computed tomography showed a necrotic lesion in the left kidney (6.5 × 5.4 cm), consistent with RCC (Fig. 1a), along with a retained CBD stent with biliary sludge, and dilated biliary ducts (Fig. 1b).

**Figure 2:**
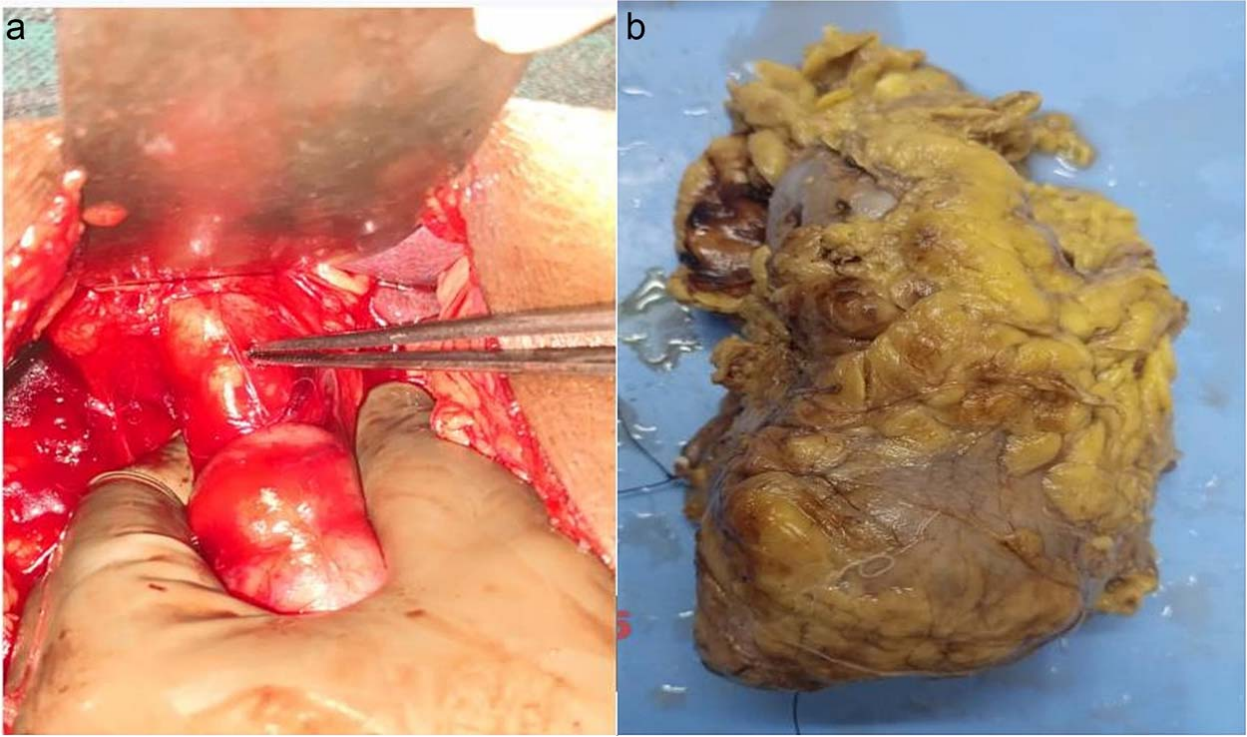
(a) Dialated CBD. (b) Formalin fixed specimen of left kidney showing mass at lower pole.

After multidisciplinary tumour board discussion, the patient planned for left radical nephrectomy and open CBD exploration through midline approach because of left RCC. The per op findings were grossly enlarged left kidney with surface protuberance present at lower pole (Fig. 2b), dense adhesions were present at right subhepatic region and grossly dilated mid & distal CBD stony hard in consistency (Fig. 2a) filled with multiple calculi (largest 3 × 2 cm), previously inserted CBD stent present. The patient underwent left radical nephrectomy along with choledocho-duodenostomy in a single sitting. The retained stent and sludge were removed successfully. The patient recovered uneventfully and was discharged on post op day 7. Histopathology confirmed chromophobe cell RCC, Fuhrman grade I (pT2aN0M0) and immunohistochemistry showed CD10 and CD117 positive along with CK7 negative. At 3-month follow-up, the patient remained asymptomatic.

## Discussion

RCC constitutes the majority of adult kidney malignancies and is often detected incidentally during imaging for unrelated conditions [1, 6]. In our case, retained CBD stent along with secondary choledocholithiasis was discovered during evaluation for the renal mass, underscoring the importance of comprehensive radiological assessment.

Choledocholithiasis, particularly when associated with long-retained plastic stents, is a known cause of recurrent cholangitis, biliary obstruction, and sludge or stone formation [3–5]. The standard recommendation is stent removal or exchange within 3–6 months to avoid complications [5]. Our patient had a stent retained for over 2 years, which likely precipitated the biliary symptoms.

RCC demands comprehensive oncological evaluation and surgical planning, whilst choledocholithiasis, especially in the context of a long-retained stent, raises the risk of biliary sepsis and infection. The need to balance urgent biliary intervention with the staging and management of a renal malignancy presents a unique clinical dilemma. There is minimal literature describing such overlapping presentations, making this case particularly notable from a diagnostic and therapeutic standpoint.

It also serves as a reminder of the importance of structured follow-up protocols and patient education regarding temporary medical devices such as biliary stents. By reporting this case, we aim to share insights on the prioritization of interventions, risks of delayed biliary stent removal, and the nuances of treating concurrent pathologies with potentially life-threatening implications. This contribution intends to raise awareness amongst clinicians about such rare but clinically significant scenarios and encourages proactive coordination across specialties for optimal patient outcomes.

The midline approach was chosen as left kidney was involved, so midline incision allowed effective access to both left kidney and hepato-biliary tract. Other reports have shown that right subcostal (for right RCC with retained CBD stent) or midline incisions provide excellent exposure for addressing pathologies involving both systems simultaneously [7, 8].

The decision for single-stage surgery was driven by patient safety, minimization of repeated anaesthesia, decrease financial burden and logistic considerations in low resource setting. Literature supports individualized approaches in dual-system pathologies, but such synchronous management requires effective coordination amongst surgical and anaesthetic teams [9, 10].

## Conclusion

This case demonstrates the practicality and safety of single-stage surgical management of concurrent RCC and retained CBD stent with secondary choledocholithiasis. It highlights the importance of structured follow-up after biliary stenting and advocates for multidisciplinary coordination in complex dual-pathology cases.
